# Fornix Mediates Information Propagation in Brain Networks Following DLPFC‐Targeted rTMS in Alzheimer's Disease: A Randomized Controlled Trial

**DOI:** 10.1111/cns.70630

**Published:** 2025-11-06

**Authors:** Yuxuan Shao, Lin Liu, Shuxiang Zhu, Ziyan Zhu, Pan Wang, Bharat B. Biswal, Hua Lin

**Affiliations:** ^1^ Department of Neurology Xuanwu Hospital, Capital Medical University Beijing China; ^2^ The Clinical Hospital of Chengdu Brain Science Institute, MOE Key Laboratory for Neuroinformation, Center for Information in Medicine, School of Life Science and Technology, University of Electronic Science and Technology of China Chengdu China; ^3^ Department of Biomedical Engineering New Jersey Institute of Technology Newark New Jersey USA

**Keywords:** Alzheimer's disease, effective connectivity, fornix, repetitive transcranial magnetic stimulation, white matter

## Abstract

**Aims:**

Repetitive transcranial magnetic stimulation (rTMS) could improve the clinical manifestations in Alzheimer's disease (AD), but its impact on deep brain tissue related to memory remains unclear. This study explored whether rTMS targeting cortical gray matter could regulate the white matter (WM) and exert modulatory effects on the network through WM bundles.

**Methods:**

Seventy‐three AD patients underwent 14‐day rTMS over the left dorsolateral prefrontal cortex (44 real, 25 sham). Granger causality analysis assessed changes in effective connectivity (EC) between the fornix and whole‐brain voxels. Furthermore, the effects of rTMS treatment on fiber tracking parameters were analyzed.

**Results:**

After rTMS therapy, patients with AD showed increased EC based on fornix in the real‐stimulation group. Functional network projections indicated that these clusters belonged to the frontoparietal network, the somatomotor network, as well as three white matter networks. Additionally, increased EC associated with fornix exhibited lateralization on the right side. Diffusion tensor imaging results showed no significant differences after the 14‐day rTMS treatment.

**Conclusion:**

In conclusion, a 14‐day rTMS treatment in AD could regulate fornical function by increasing cortical‐fornix EC, indicating neuroplasticity changes in response to therapy.

**Trial Registration:**

Chinese Clinical Trial Registry (https://www.chictr.org.cn/index.html; ChiCTR2200062564)

## Graphical abstract

We enrolled Alzheimer's disease participants who received 14‐day repetitive transcranial magnetic stimulation (rTMS) over the left dorsolateral prefrontal cortex (44 real rTMS, 25 sham). Real rTMS enhanced fornix‐based connectivity to frontoparietal, somatomotor, and white matter networks, highlighting the fornix as a mediator and potential biomarker of therapeutic response.

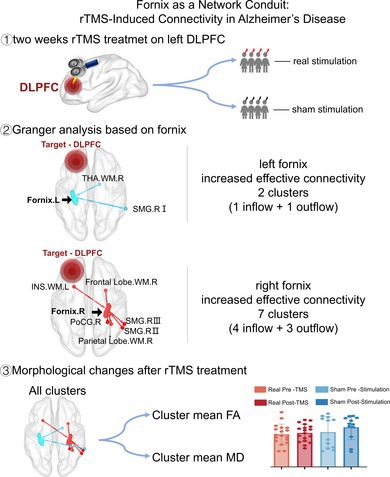

## 1 Introduction

Alzheimer's disease (AD) is a progressive neurodegenerative disease characterized by the deposition of amyloid‐beta (Aβ) peptides and neurofibrillary tangles, resulting in abnormal brain electrical activity [1]. As a noninvasive therapy, repetitive transcranial magnetic stimulation (rTMS) can rapidly change magnetic fields to modulate the electrical activity in the human brain [2]. Cognitive functions in individuals with AD have been improved by adopting rTMS treatment technology to stimulate specific cortical areas. For example, rTMS therapy targeting the left angular gyrus in AD induced functional changes in the default mode network [3]. Another study involving in‐patients with mild cognitive impairment showed functional changes in the salience network and the frontoparietal network after rTMS treatment on the bilateral left dorsolateral prefrontal cortex (DLPFC) [4]. It remains unknown whether rTMS targeting cortical gray matter (GM) could regulate the white matter (WM) and exert modulatory effects on the network through WM bundles.

As the important WM bundle pathway in the human brain's memory circuit, the bilateral fornix modulates cognition and episodic memory recall [5, 6]. This essential pathway originates from the bilateral hippocampi, merging at the midline of the brain and crossing to the contralateral side [7]. Patients with fornix damage typically exhibit severe deficits in learning and memory [8], further demonstrating its importance in memory‐related cognitive functions. Previous studies have reported that fornix atrophy can effectively predict the onset of AD before clinical symptoms become apparent [9, 10]. Additionally, as an important target for deep brain stimulation in AD, the phase I and II trials of deep brain stimulation targeting the fornix can effectively improve patients' memory and other cognitive functions [11, 12]. However, deep brain stimulation is an invasive treatment with high surgical risks. Therefore, rTMS has shown unique advantages in treating AD compared to deep brain stimulation treatment, but how it regulates the memory‐related fornix tissue has not been investigated.

Accumulated evidence demonstrated that the blood‐oxygen‐level‐dependent (BOLD) signals in WM can be reliably detected during both resting and various task activation states [13]. WM BOLD signals are not noise, as previously thought, but rather have a physiological function [14, 15, 16]. Moreover, recent studies on WM have characterized pathological functional changes in several neuropsychiatric disorders, including schizophrenia [17], Parkinson's disease [18], and AD [19]. Our previous study reported that rTMS targeted at the angular gyrus in AD led to increased functional connectivity between the angular gyrus and activated WM regions [20]. Given the crucial role of fornix as a deep WM bundle related to the memory circuit, it is important to detect the functional changes of fornix following rTMS targeting superficial cortical GM.

Compared to functional connectivity, effective connectivity further provides directional information between two remote areas that are functionally connected [21]. Analyzing the effective connectivity changes between WM and whole‐brain other voxels after rTMS therapy would promote an understanding of the rTMS modulatory effect on WM function. Considering the directional nature of effective connectivity, Granger causality analysis (GCA) is generally used on resting‐state functional magnetic resonance imaging (fMRI) data to investigate the positive or negative influences of stimulation targets on the rest of the brain following rTMS stimulation [22]. GCA has been employed to assess brain activity in various diseases, such as attention‐deficit/hyperactivity disorder [23], frontal lobe epilepsy [24], and stroke [25]. Additionally, patients with Parkinson's disease exhibited attenuated interactions of the WM functional networks using GCA, which were associated with Parkinson's symptoms [18].

We hypothesized that rTMS treatment would produce a modulatory effect on the fornix to influence cortical activity and cognitive function in AD. To this end, we collected fMRI data before and after rTMS treatment in AD to investigate the effective connectivity changes based on the bilateral fornix as the seed point. Moreover, we investigated the fornix‐whole brain effective connectivity in both real and sham groups and estimated the relationships between clinical symptoms, improved scores, and neuroimaging alterations. We further compared diffusion tensor imaging (DTI) parameters before and after the treatment to identify structural plasticity changes in the brain induced by rTMS.

## 2 Materials and Methods

### 2.1 Study Design

The study design was triple‐blind, randomized, and sham‐controlled. Participants were randomized to either the real or sham rTMS treatment group at a 1:1 ratio according to a computer‐generated randomization table. The primary outcome was the improvement in clinical scale scores after treatment in the real stimulation group.

### 2.2 Participants' Information

From May 2021 to October 2023, 73 AD patients were enrolled; 48 received real rTMS targeting the left DLPFC, and 25 received sham stimulation. Four real‐stimulation patients were excluded (3 for excessive head motion, 1 for age matching), leaving 69 (44 real rTMS stimulation, 25 sham). The inclusion criteria for the study were: (i) probable Alzheimer's dementia diagnosis according to the 2011 National Institute on Aging AD (NIA‐AA) guidelines; (ii) Clinical Dementia Rating score of 0.5 or 1.0; (iii) Mini‐Mental State Examination (MMSE) score less than 27; (iv) age range from 55 to 80 years and right‐handedness; (v) stable dose of donepezil for at least 3 months prior to intermittent theta‐burst stimulation (iTBS) intervention, continuing until follow‐up completion. The exclusion criteria included: (i) clinical features suggestive of a pathology other than AD; (ii) history of other neurological or severe systemic diseases; (iii) personal (or first‐degree relative) history of seizures; (iv) current use of benzodiazepines or a history of substance abuse; (v) presence of focal brain lesions on T1 or T2 images; (vi) non‐MRI compatible implanted devices such as pacemakers, deep brain stimulators, aneurysm clips, or coronary stents.

### 2.3 Ethical Statement

All participants were recruited from Xuanwu Hospital, Capital Medical University, China, and signed informed consent. Clinical diagnoses were assessed by senior neurologists. The study was approved by the Medical Research Ethics Committee of Xuanwu Hospital, Capital Medical University, China, and registered on the site Chinese Clinical Trial Registry (https://www.chictr.org.cn/index.html).

### 2.4 Neuroimaging Acquisition

Over two scanning days, we conducted baseline and post‐treatment scans, collecting resting‐state fMRI, T1‐weighted anatomical images, and diffusion tensor images using a clinical Siemens MAGNETOM Skyra 3.0 T MRI system (Siemens, Erlangen, Germany). Resting‐state fMRI was acquired using a gradient echo EPI sequence in two batches: First batch: Repetition time (TR) = 2000 ms, echo time (TE) = 30 ms, flip angle = 90°, 32 contiguous slices, voxel size = 3.5 × 3.5 × 3.0 mm^3^; Second batch: TR = 3000 ms, TE = 30 ms, flip angle = 90°, 32 contiguous slices, voxel size = 3.5 × 3.5 × 4.375 mm^3^. During resting‐state fMRI scanning, patients were instructed to fixate on a white cross centered on a screen with a black background in a low‐luminance environment. Anatomical sagittal T1‐weighted MP‐RAGE images were obtained using: TR = 2530 ms, TE = 2.98 ms, flip angle = 7°, voxel size = 1.0 × 1.0 × 1.0 mm^3^. The DTI dataset was acquired using an EPI sequence with 64 directions, slice thickness = 2 mm, TR = 11,800 ms, TE = 87 ms, flip angle = 90°, b = 1000 s/mm^2^.

### 2.5 Transcranial Magnetic Stimulation Parameters

**FIGURE 1:**
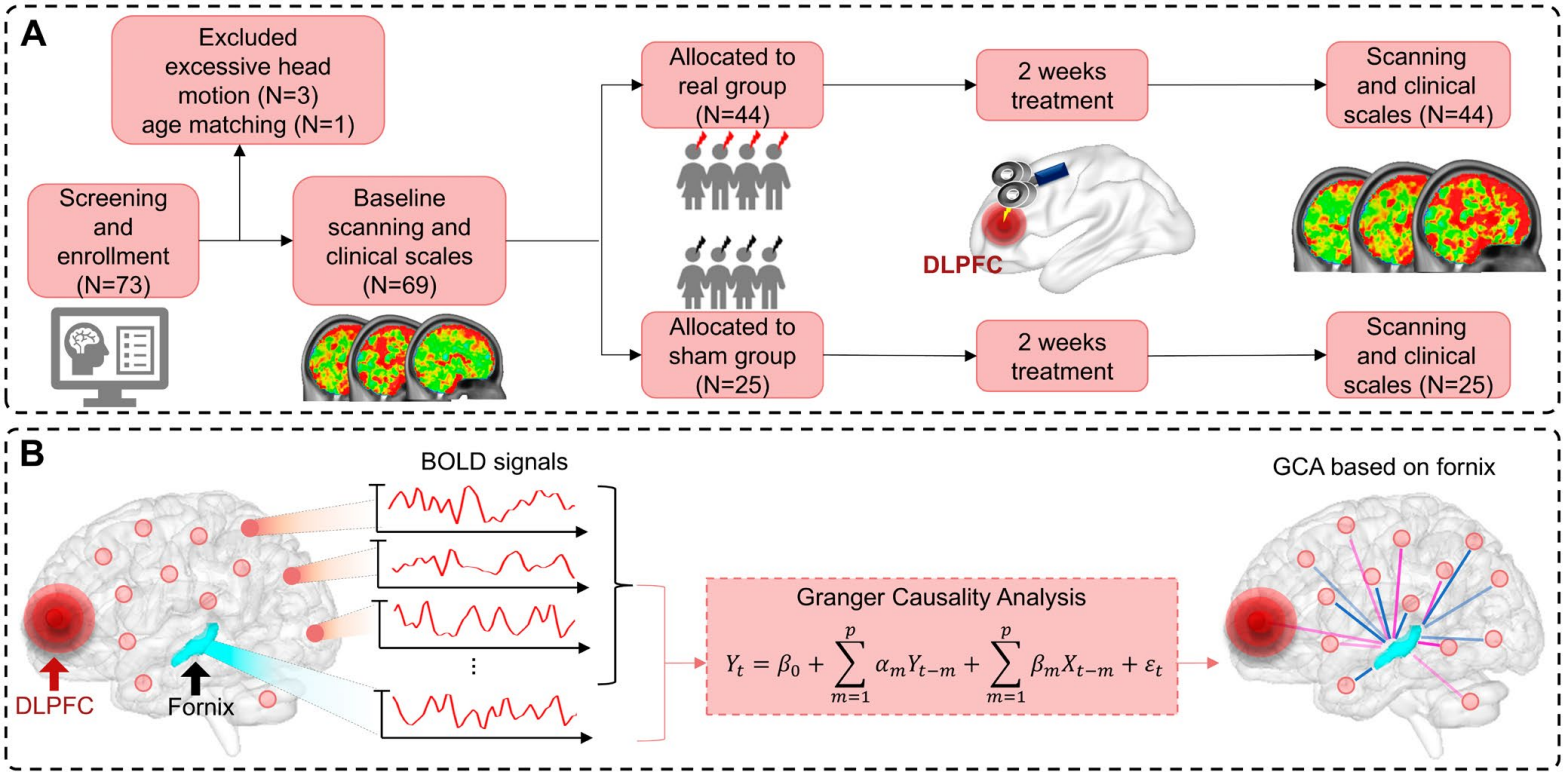
The flow chart of the study. (A) The process diagram of data acquisition and rTMS treatment. (B) Granger causality analysis between the whole‐brain voxels and fornix.

An accelerated high‐dose iTBS protocol was performed using a Magstim Rapid2 transcranial magnetic stimulator (Magstim Co. Ltd., UK) with an air‐cooled figure‐eight coil (70 mm diameter). The left DLPFC (Montreal Neurological Institute (MNI) coordinates [−38 44 26]) was targeted based on individual structural MRIs and guided by a Brainsight TMS neuro‐navigation system (Brainsight, Rogue Resolutions Inc., Canada). The protocol included 28 iTBS sessions over 14 days (two sessions per day with a 50‐min interval), delivering 50,400 pulses in total. Each session consisted of 1800 pulses in 60 triplet bursts, with a 2 s train duration and 8 s cycling period, at 80% of the resting motor threshold. The sham intervention used a Magstim pseudo‐stimulus coil placed over the left DLPFC, identical in appearance to the real coil, mimicking scalp sensations and acoustic artifacts but delivering no actual stimulation. The sham group received identical parameters and schedule as the real group (Figure 1A).

### 2.6 Preprocessing Procedures for fMRI Data

#### 2.6.1 Functional Images

We conducted the preprocessed analysis by using the Data Processing Assistant for Resting‐State fMRI (http://rfmri.org/DPARSF) and SPM12 software (http://www.fil.ion.ucl.ac.uk/spm/software/spm12). We discarded the first 10 time points and performed the slice‐timing and head motion correction. The maximum displacements of all subjects were less than 3 mm or 3°. The structural image for each participant was co‐registered to its corresponding functional image, and each structural image was segmented into GM, WM, and cerebrospinal fluid. We performed the removal of linear trends to allow for the correction of signal drift and nuisance covariates regression using Friston 24 head motion parameters and averaged the CSF signal. Filtering with a range from 0.01 to 0.1 Hz was used to reduce the noise from non‐neuronal activity. To avoid mixing WM and GM signals due to partial‐volume effect, functional images were minimally spatially smoothed (4 mm full‐width half‐maximum, isotropic) separately within WM and GM templates for each participant. Following smoothing, functional images were transformed from individual native space into MNI space in 3 × 3 × 3 mm^3^.

#### 2.6.2 Diffusion Tensor Images

We preprocessed and analyzed the DTI data using the FSL software (http://www.fmrib.ox.ac.uk/fsl). Eddy currents and subject motion were estimated and corrected using the FSL eddy tool. This procedure uses a Gaussian process to simultaneously model the effects of eddy currents and head motion on diffusion‐weighted volumes, resampling the data only once. Diffusion gradient vectors were also rotated to adjust for subject motion estimated by eddy. For each subject, we obtained the voxel‐level fractional anisotropy (FA) and mean diffusivity (MD) to estimate the effects of rTMS on brain structural plasticity. The formulas for these two parameters are as follows:

$$\mathrm{FA}=\frac{\sqrt{3\left({\left(\lambda_{1}-\bar{\lambda }\right)}^{2}+{\left(\lambda_{2}-\bar{\lambda }\right)}^{2}+{\left(\lambda_{3}-\bar{\lambda }\right)}^{2}\right)}}{\sqrt{2\left(\lambda_{1}^{2}+\lambda_{2}^{2}+\lambda_{3}^{2}\right)}}$$

$$\mathrm{MD}=\frac{\lambda_{1}+\lambda_{2}+\lambda_{3}}{3}$$

where $\lambda_{1}$, $\lambda_{2}$, $\lambda_{3}$ are the eigenvalues of the diffusion tensor, representing the diffusivity of water molecules in the brain at different direction, $\lambda_{1}$ is the diffusion parallel to the axon direction, $\lambda_{2}$ and $\lambda_{3}$ are the direction perpendicular to $\lambda_{1}$.

After preprocessing the DTI data, we normalized the above FA and MD maps of all subjects from individual space to MNI space using the following procedures: (i) The T1 images were co‐registered to the B0 images; (ii) The co‐registered T1 images were normalized to the MNI space, and the transformation matrices were then obtained; (iii) The FA and MD maps in individual space were warped to the MNI space using the transformation matrices defined above. Furthermore, we performed paired *T*‐tests and two‐sample *T*‐tests to explore the differences between four groups (pre‐rTMS, post‐rTMS, pre‐sham stimulation, and post‐sham stimulation). Then, we analyzed the differences in DTI parameters in the fornix.

### 2.7 Granger Causality Analysis Based on the Fornix

The GCA method allows us to assess causality among two signals, in which one signal, *Y*, is said to granger cause another signal, *X* if the past of *Y* and *X* can better predict the future of *X* rather than with the past of *X* only [26]. To evaluate the regulatory effect of the fornix on the cortex, we used this principle to investigate the pairwise multivariate conditional Granger causalities of our independent components. We performed the GCA between fornix and whole‐brain voxels adopting the DynamicBC toolkit [27]. The autoregressive model is defined as follows:

$$Y_{t}=\beta_{0}+\sum_{m=1}^{p}\alpha_{m}Y_{t-m}+\sum_{m=1}^{p}\beta_{m}X_{t-m}+\varepsilon_{t}$$

where $X_{t}$ and $Y_{t}$ are BOLD time series of two regions with causal relationships detected, *t* is the current time points, $\alpha_{m}$ and $\beta_{m}$ are the linear prediction coefficients for *Y* and *X*, $\varepsilon_{m}$ is the residual error, *p* is the lag time, when *p* = 1 is the autoregressive model order. Assuming that variable *X* is not the cause of change in variable *Y*, so $\beta_{m}$ is all 0, A simplified autoregressive model with constraints is constructed. Finally, *F* statistics is performed on comparing the residual variance of the autoregressive model and the simplified autoregressive model to constructed the significance level of bivariate GCA. A positive GCA value in *X* to *Y* suggests that *X* causes a statistical change in *Y*, and it is referred as a probable directional relationship in *X*‐to‐*Y*.

We extracted the fornix mask from the JHU ICBM‐DTI‐81 WM atlas [28], and separately selected both the left and right fornix as seed regions. We defined the GCA from the seed region to the whole‐brain voxels as the outflow and from the whole‐brain voxels to the seed region as the inflow (Figure 1B). We calculated the fornix–whole brain causality for outflow and inflow to identify the causal effect between the fornix and whole‐brain voxels.

### 2.8 Statistical Analysis

The sample size was determined by setting the type I error at 0.05 (two‐sided). Using the two independent samples difference test module in PASS 11, the required sample size was calculated to be 40 participants per group. Considering a 10% dropout rate, the final sample size was adjusted to 50 participants per group, resulting in a total of 100 participants. For demographic analyses, sex, as a categorical variable, was compared using the chi‐square test. Age, as a continuous variable, was first subjected to the Shapiro–Wilk test for normality within the real and sham groups, both of which followed a normal distribution (real group: *W* = 0.984, *p* = 0.736; sham group: *W* = 0.965, *p* = 0.441). Therefore, an independent‐samples *t*‐test was used to compare age between groups (*p* = 0.068, not significant). Years of education did not conform to normality and were analyzed using the non‐parametric Mann–Whitney test. For neuropsychological scales (MMSE, AVLT, MoCA), given their ordinal nature, normality testing was not applied; instead, the Wilcoxon signed‐rank test was used to assess distribution differences between baseline and post‐treatment scores.

Regarding neuroimaging data, whole‐brain voxel‐wise GCA was conducted to examine four causal directions: cortical voxels inflow into the left fornix, left fornix outflow to cortical voxels, cortical voxels inflow into the right fornix, and right fornix outflow to cortical voxels. During preprocessing, GCA data were standardized and spatially smoothed to approximate normality, and voxel‐wise statistics in SPM12 rely on large‐sample Gaussian assumptions. Thus, paired *t*‐tests were applied to assess within‐group changes (pre vs. post for real and sham rTMS groups). For the GCA brain maps, voxel‐wise paired *t*‐tests in SPM12 yielded statistical outputs in terms of *t*‐ and *p*‐values, without direct estimates of statistical power or confidence intervals (CIs). We obtained the statistical effect map on Cohen's *d* for the paired *t*‐test analysis of GCA (Figure S1). Gaussian Random‐Field (GRF) correction was employed for multiple comparison correction using a two‐tailed test (voxel level *p* < 0.01, cluster level *p* < 0.05). DTI data did not meet the assumption of normality; therefore, all statistical comparisons were performed using non‐parametric methods (Mann–Whitney test and Wilcoxon signed‐rank test).

## 3 Results

### 3.1 Clinical Results

Forty‐four patients received real rTMS treatment targeting the left DLPFC, and the remaining twenty‐five patients received sham stimulation. For each participant, we used the scales for AVLT, MMSE, and MoCA scores to estimate patients' cognitive function. Their detailed clinical and demographic information is described in Table 1. After treatment, the real rTMS group showed significant improvements in AVLT, MMSE, and MoCA scores compared with the baseline. In the sham stimulation group, the outcome scores were also examined, and statistical testing indicated changes after treatment.

**TABLE 1:** Demographic and clinical characteristics of subjects.

Characteristic	Real TMS group		Sham stimulation group		Comparison	
	Pre‐rTMS	Post‐rTMS	Pre‐STIM	Post‐STIM	Real pre/post	Sham pre/post
Number	44		25		—	—
Age	68.64 ± 6.41		65.52 ± 7.17		0.112^b^	
Gender (M/F)	14/30		6/19		0.491^c^	
Education	11.68 ± 3.08		11.32 ± 2.91		0.765^b^	
AVLT	19.50 ± 7.43	24.52 ± 10.00	22.12 ± 7.55	27.00 ± 7.81	< 0.001^a^	< 0.001^a^
MMSE	25.45 ± 3.66	26.61 ± 3.37	25.36 ± 4.13	27.16 ± 3.83	< 0.001^a^	< 0.001^a^
MoCA	21.18 ± 4.45	23.11 ± 5.18	20.84 ± 4.47	23.84 ± 4.47	< 0.001^a^	< 0.001^a^

Abbreviations: AVLT, auditory verbal learning test; F, female; M, male; MMSE, Mini‐Mental State Examination; MoCA, montreal cognitive assessment; STIM, stimulation; TMS, transcranial magnetic stimulation.

^a^ Wilcoxon matched‐pairs signed rank test.

^b^ Mann–Whitney test.

^c^ Chi‐Square Test.

### 3.2 Neuroregulatory Effects of Stimulation on Fornix

**FIGURE 2:**
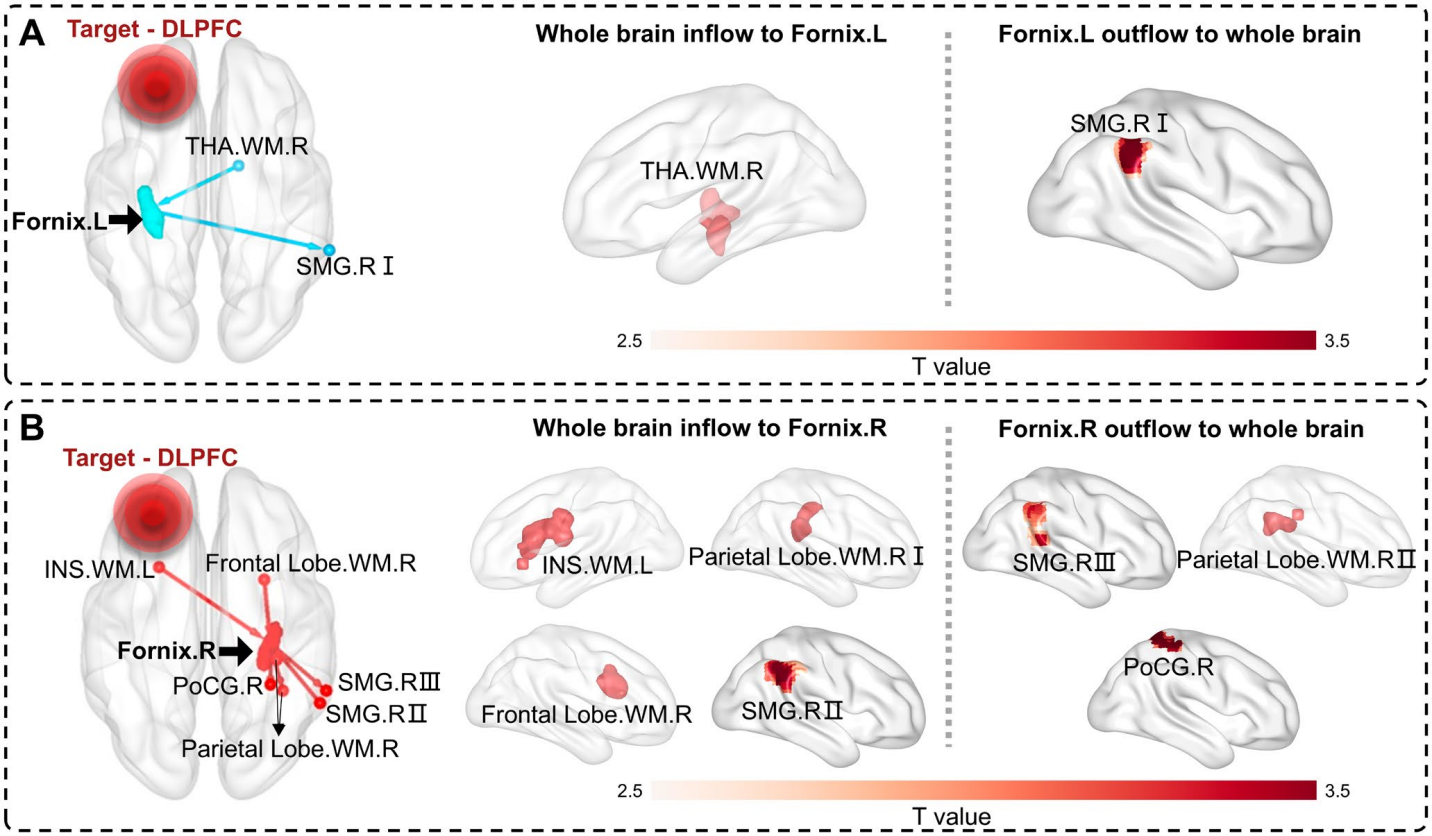
The pair *t*‐test results of before and after rTMS treatment in the real‐stimulation group. (A) The GCA modulation route on the left fornix. (B) The GCA modulation route on the right fornix. GRF correction is performed using a two‐tailed test to estimate statistical significance, with voxel‐level *p* < 0.01 and cluster‐level *p* < 0.05. Color bars are proportional to *T* values. The left and right fornix are marked as blue and red, respectively. DLPFC, dorsolateral prefrontal cortex; Frontal Lobe.WM.R, the right frontal lobe in WM; INS.WM.L, the left insula in WM; Parietal Lobe.WM.R, the right parietal lobe in WM; PoCG.R, the right postcentral cortex; SMG.R, the partial right supramarginal region; THA.WM.R, the right thalamus in WM.

To investigate the effective connectivity changes after rTMS treatment, we analyzed the GCA effects of stimulation on the fornix. Within‐group analysis exhibited a robust distribution pattern in four groups. Besides, GCA results from the pair *t*‐test between pre and post‐rTMS showed GCA changes for the bilateral fornix in the real‐stimulation group. Specifically, the left fornix was affected by the right thalamus in WM, and it also modulated the partial right supramarginal region I (Figure 2A). Compared to the pre‐rTMS group, participants in the real group showed increased outflow modulations between the right fornix and right supramarginal region III, right parietal lobe in WM II and right postcentral cortex. Additionally, we observed increased inflow modulations between the right fornix and left insula in WM, right parietal lobe in WM I, right frontal lobe in WM, and right supramarginal gyrus II (Figure 2B).

To investigate whether rTMS targeting cortical GM (node) exerts its modulatory effects on the network through WM bundles (edge), we examined the functional network projections in the real‐stimulation group. The connections between the fornix and whole‐brain voxels are mainly reflected, and detailed cluster information is described in Table 2. Finally, we did not observe significant GCA changes on the fornix before and after treatment in the sham stimulation group.

**TABLE 2:** Information of significantly different clusters.

Group	Brain regions	Distributed network	Cluster size (voxels)	Peak intensity (*T*)	MNI coordination		
					*x*	*y*	*z*
Fornix‐L‐OUT	SMG.RI	FPN	86	3.7745	60	−42	39
Fornix‐R‐IN	SMG.RII	FPN	177	3.8456	54	−51	42
Fornix‐R‐OUT	SMG.RIII	FPN	107	3.568	57	−45	48
	PoCG.R	SMN	83	4.2529	30	−42	63
Fornix‐L‐IN	THA.WM.R	K12‐8	206	4.1949	15	0	−6
Fornix‐R‐IN	INS.WM.L	K12‐11	213	4.4835	−24	15	12
	Frontal Lobe.WM.R	K12‐11	99	4.1444	27	9	21
	Parietal Lobe.WM.RI	K12‐5	102	4.0371	33	−30	24
Fornix‐R‐OUT	Parietal Lobe.WM.RII	K12‐5	83	4.0425	36	−45	33

Abbreviations: FPN, frontoparietal network; Frontal Lobe.WM.R, the right frontal lobe in WM; INS.WM.L, the left insula in WM; K12‐11, Deep frontal white matter; K12‐5, superior longitudinal fasciculus system; K12‐8, inferior corticospinal tract; K12‐i, the i network in K12; Parietal Lobe.WM.R, the right parietal lobe in WM; PoCG.R, the right postcentral cortex; SMG.R, the partial right supramarginal region; SMN, the Somatomotor network; THA.WM.R, the right thalamus in WM.

### 3.3 Lateralization of Effective Connectivity Associated With Fornix

**FIGURE 3:**
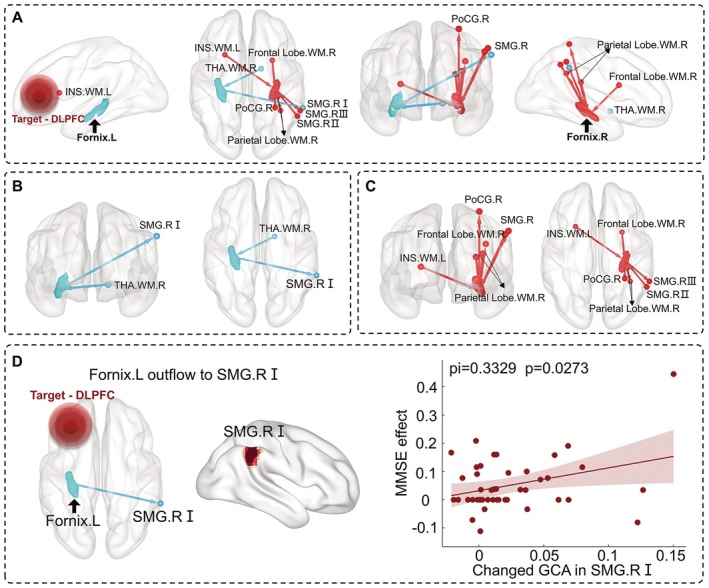
Lateralization of EC associated with fornix and correlation analysis with therapeutic effect. (A) Whole‐brain EC related to left and right fornix. (B) and (C) respectively represent the whole‐brain EC related to the left and right fornix. (D) The changed GCA value with left fornix modulating SMG.RI had a significantly positive correlation with the therapeutic effect represented by MMSE (*r* = 0.3329, *p* = 0.0273). DLPFC, dorsolateral prefrontal cortex; EC, effective connectivity; Frontal Lobe.WM.R, the right frontal lobe in WM; INS.WM.L, the left insula in WM; Parietal Lobe.WM.R, the right parietal lobe in WM; PoCG.R, the right postcentral cortex; SMG.R, the partial right supramarginal region; THA.WM.R, the right thalamus in WM; Treatment effect, the post‐treatment scores minus the pretreatment scores and then divided by the pretreatment scores and multiplied by 100%.

To investigate the differences in the modulation of the bilateral fornix by rTMS, we extracted the location of each cluster's peak *t*‐value point. We observed one cluster flowing into the left fornix, one cluster out, three clusters flowing into the right fornix, and four clusters out (Figure 3A). Specifically, the left fornix affected the partial right supramarginal region I in the right hemisphere, and the right thalamus in WM modulated the left fornix (Figure 3B). Additionally, the right fornix affects the partial right supramarginal region III, the right parietal lobe in WM II, the right postcentral cortex in the right hemisphere, and the right parietal lobe in WM I, the right frontal lobe in WM, and the partial right supramarginal region II modulated the right fornix. Only the left insula in WM in the left hemisphere modulated the right fornix (Figure 3C).

### 3.4 Correlations Between Effective Connectivity Changes and Clinical Scores

To investigate the relationships between the above‐mentioned abnormal clusters and clinical symptoms, we conducted Pearson's correlation analyses between them, controlling for age, gender, and education. We observed a positive correlation between the changed effective connectivity values of the left fornix modulating the partial right supramarginal region I and the therapeutic effect represented by MMSE (*r* = 0.3329, *p* = 0.0273) (Figure 3D).

### 3.5 Effects of Short‐Term Stimulations on Morphological Anatomical Parameters

**FIGURE 4:**
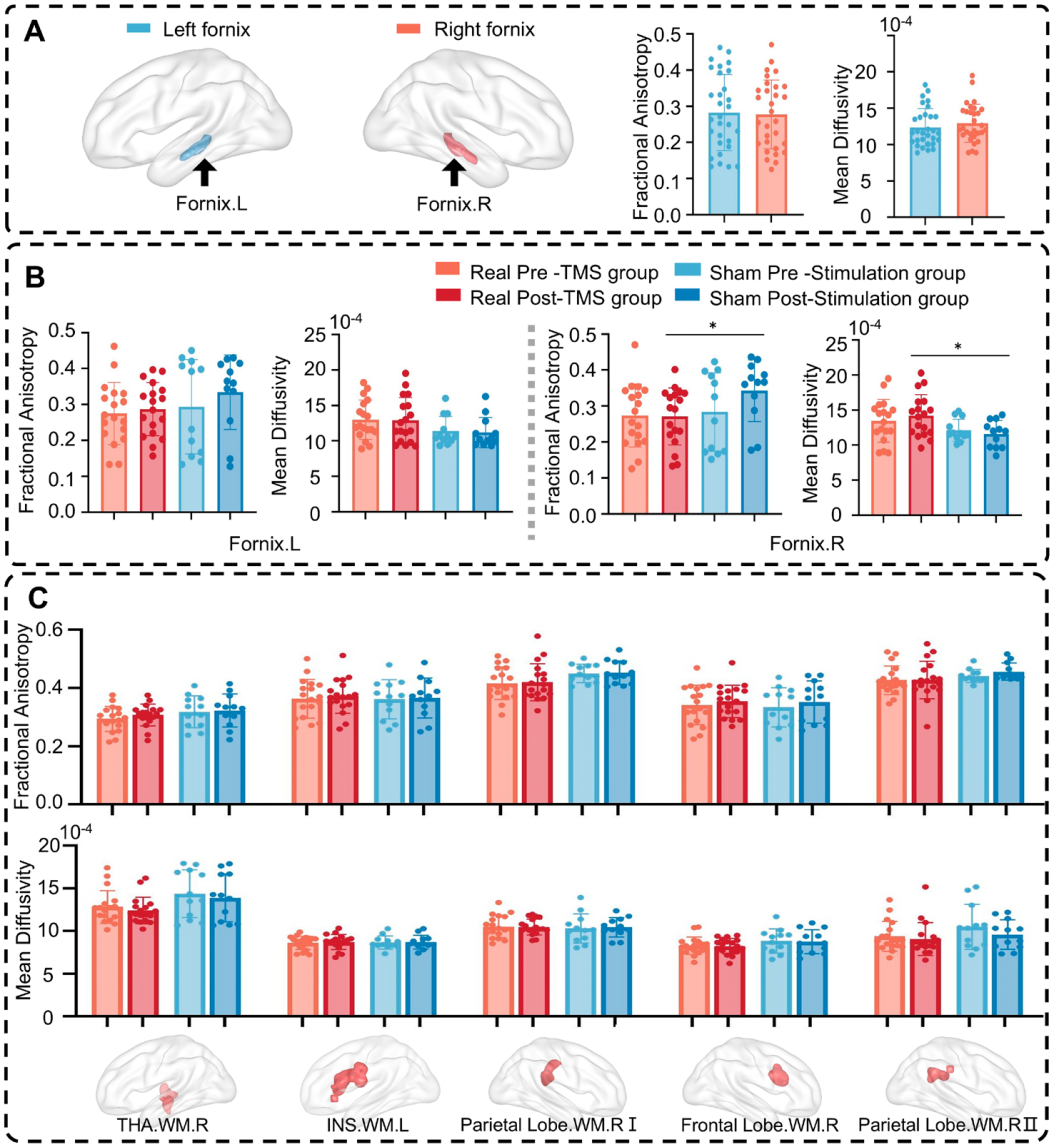
Morphological changes after rTMS treatment in AD. (A) FA and MD values for bilateral fornix in the baseline period. (B) FA and MD difference analysis for the bilateral fornix before and after rTMS treatment. (C) Morphological changes analysis for changed WM clusters modulated by rTMS treatment. FA, fractional anisotropy; Frontal Lobe.WM.R, the right frontal lobe in WM; INS.WM.L, the left insula in WM; MD, mean diffusivity; Parietal Lobe.WM.R, the right parietal lobe in WM; THA.WM.R, the right thalamus in WM; The symbol * indicates *p* < 0.05 without correction.

To explore whether rTMS treatment induces changes in structural plasticity in AD, we performed DTI analysis and further explored the differences before and after rTMS treatment (Figure 4A). Group‐level within‐template analyses of FA and MD values revealed only minimal differences between baseline and post‐treatment in the rTMS group, and no differences in the sham group. Moreover, paired *t*‐tests comparing pre‐ and post‐treatment DTI metrics did not yield significant results, indicating that no detectable changes in DTI measures were present in either the rTMS or sham groups (Figure S2). Neither the real‐stimulation group nor the sham‐stimulation group showed significant changes in DTI parameters before and after rTMS therapy (Figure 4B). Additionally, we did not observe DTI changes in the functionally altered WM clusters modulated by rTMS (Figure 4C). For the right fornix, the FA and MD measures exhibited the following statistical characteristics: right fornix FA, statistical power = 0.82, effect size *d* = −0.91, 95% confidence interval [−0.142, −0.014]; right fornix MD, statistical power = 0.88, effect size *d* = 0.94, 95% confidence interval [0.000077, 0.000442].

## 4 Discussion

The study investigated whether rTMS treatment in AD exerts its modulatory effects on the deep brain WM bundles associated with memory function. Our results demonstrated that rTMS regulated the fornical function, especially on the right side with increasing cortical‐fornix effective connectivity after rTMS treatment. Network projections showed that rTMS influenced the information integration in some GM functional networks, including the frontoparietal network and the somatomotor network, as well as in WM functional networks corresponding to the superior longitudinal fasciculus system, the inferior corticospinal tract, and the deep frontal WM. Additionally, we did not observe significant structural changes after 14‐day rTMS treatment in AD. Our findings provided new insights into the mechanisms by which rTMS modulates the deep WM bundle related to memory function, emphasizing the important role of WM bundles in the propagation of rTMS effects.

The rTMS therapy in AD increased the regulatory effect of the fornix on the cortex, distributing in the partial right supramarginal region, the right postcentral cortex, the right thalamus in WM, the left insula in WM, the right frontal lobe in WM, and the right parietal lobe in WM. Previous studies showed structural impairments of the fornix [29] as well as decreased functional connectivity between the fornix and distant brain regions in AD [30]. Our study indicated that rTMS could repair impaired function of the fornix in AD by increasing cortical‐fornix effective connectivity. Animal models suggest rTMS promotes the survival and maturation of newborn oligodendrocytes and enhances myelination [31], leading to alterations in WM [32], supporting the biological basis for rTMS modulation of WM bundles. Moreover, our study not only focused on rTMS modulation of GM functional networks but also on the WM network, revealing distinct differences in the modulatory effects on brain networks. Compared to GM networks, WM clusters were more easily modulated by rTMS. The DLPFC and the supramarginal gyrus are critical GM nodes of the frontoparietal network associated with working memory, attention, and decision‐making [33]. The supramarginal gyrus plays a crucial role in processing phonological inputs and outputs, working memory, sustained attention, task control, and other cognitive functions [34]. We found that the improvement rate in MMSE scores was positively correlated with the increased effective connectivity from the left fornix to the right supramarginal gyrus, indicating that rTMS could improve patients' cognitive performance by enhancing the effective connectivity between cortical GM and deep WM. However, improvements in clinical scores were observed in both real and sham groups, which may be associated with the placebo effect in the sham group. With significantly increased effective connectivity to the fornix, the three WM networks corresponding to the superior longitudinal fasciculus, the inferior corticospinal tract, and the deep frontal WM can be organized into deep, superficial, and middle layers [35]. Therefore, rTMS therapy in AD modulated the brain networks by increasing effective connectivity between the frontoparietal network and the fornix and between the fornix and different layers of WM networks, enabling these brain networks to cooperate more efficiently with the DLPFC through the fornix. Our results indicated that WM plays an important role as GM in rTMS neuromodulation to repair brain networks in AD.

We further confirmed that rTMS treatment targeting the left DLPFC resulted in functional improvements in the bilateral fornix, particularly on the right side. The right fornix had more connections to other areas than the left. Structural differences between the left and right fornix before treatment were not found. Thus, the lateralization effect of rTMS is not due to the variability in the fornix structures in AD. The supramarginal gyrus and postcentral gyrus are in the posterior parietal cortex. Structural connectivity supports the hypothesis that rTMS on the left DLPFC could modulate the bilateral fornix, based on the theory that structural connectivity underpins functional connectivity [36, 37, 38]. AD is a syndrome of disconnection, with disrupted brain network connectivity [39, 40]. The left hemisphere is more severely affected and exhibits a faster degeneration rate in AD [41]. Yang and colleagues revealed disease‐related disruptions in the efficiency of the WM network in the left hemisphere, while only slight changes were observed in the right [42]. We speculated that rTMS on the left DLPFC showed lateralized modulation efficacy due to the functional asymmetry of the bilateral fornix in AD, with the right fornix being more responsive due to its more efficient communication.

After the 14‐day rTMS treatment, DTI metrics revealed no structural neuroplasticity changes, whereas fMRI indicated functional neuroplasticity changes. Our results are inconsistent with several reports. For example, Peng et al. found that 4 weeks of high‐frequency rTMS increased FA values of the WM tracts in treatment‐resistant depression patients [43]. Additionally, Brabenec and colleagues found increased WM integrity within the auditory‐motor loop in Parkinson's patients during a 2‐month follow‐up after 2 weeks of rTMS [32]. The discrepancy may be due to different rTMS protocols, treatment duration, and disease heterogeneity. Structural changes after rTMS may relate to long‐term enhancement from multiple sessions, altering synaptic plasticity and leading to lasting structural changes. Studies using fMRI and/or DTI to analyze brain connectivity have produced inconsistent results, possibly due to the different sensitivities of these imaging modalities [19]. The sensitivity comparison between fMRI and DTI remains unclear. Our findings indicated that fMRI might be a more sensitive biomarker for evaluating the efficacy and brain plasticity of rTMS, further suggesting that a short‐term 14‐day rTMS treatment could induce functional neuroplasticity changes in AD. Extending rTMS treatment duration holds promise for inducing structural neuroplasticity changes in AD.

Several limitations should be noted. First, the sham group had a smaller sample size than the real treatment group, possibly contributing to a large placebo effect. Second, likely due to the short treatment period, no aftereffects on DTI were observed. It would be interesting to estimate whether repeating the iTBS protocol every 2 months could reveal DTI changes. Third, subjects were included based on clinical rather than pathological diagnoses. Future studies with broader enrollment criteria could help reveal the effects of rTMS and validate our findings.

## 5 Conclusion

The study demonstrated that rTMS targeting left DLPFC had modulatory effects on cortical‐fornix connections in AD. As the key pathway in the human brain's memory circuit and an important target for deep brain stimulation, the fornix plays a crucial role in disseminating the rTMS effect from the targeted region to distant areas. Additionally, we found a right lateralization effect of rTMS on the regulation of the fornix and did not observe significant anatomical changes after the 14‐day rTMS treatment. Our findings indicated that rTMS treatment could rewire brain networks by increasing cortical‐fornix effective connectivity in AD and inducing changes in functional neuroplasticity, providing new insights into the mechanisms of rTMS therapy.

## Author Contributions

Y.S.: investigation, writing original draft, writing – review and editing; L.L.: data curation, visualization, writing original draft, writing – review and editing; S.Z.: investigation; Z.Z.: investigation; P.W.: formal analysis, methodology, supervision, writing – review and editing; B.B.B.: supervision, writing‐review and editing; H.L.: conceptualization, project administration, supervision, writing‐review and editing. All authors have approved the final manuscript.

## Conflicts of Interest

The authors declare no conflicts of interest.

## Supporting information


**Data S1:** Supporting Information.

## Data Availability

The data that support the findings of this study are not openly available due to reasons of sensitivity and are available from the corresponding author upon reasonable request.
