# Supplementary material for: Fornix Mediates Information Propagation in Brain Networks Following DLPFC‐Targeted rTMS in Alzheimer's Disease: A Randomized Controlled Trial
Source: CNS Neurosci Ther. 2025 Nov 6;31(11):e70630. doi: 10.1111/cns.70630 (PMC12592100; doi:10.1111/cns.70630)
Supplement: Supplementary file 1 — Data S1: Supporting Information. [file CNS-31-e70630-s001.docx]

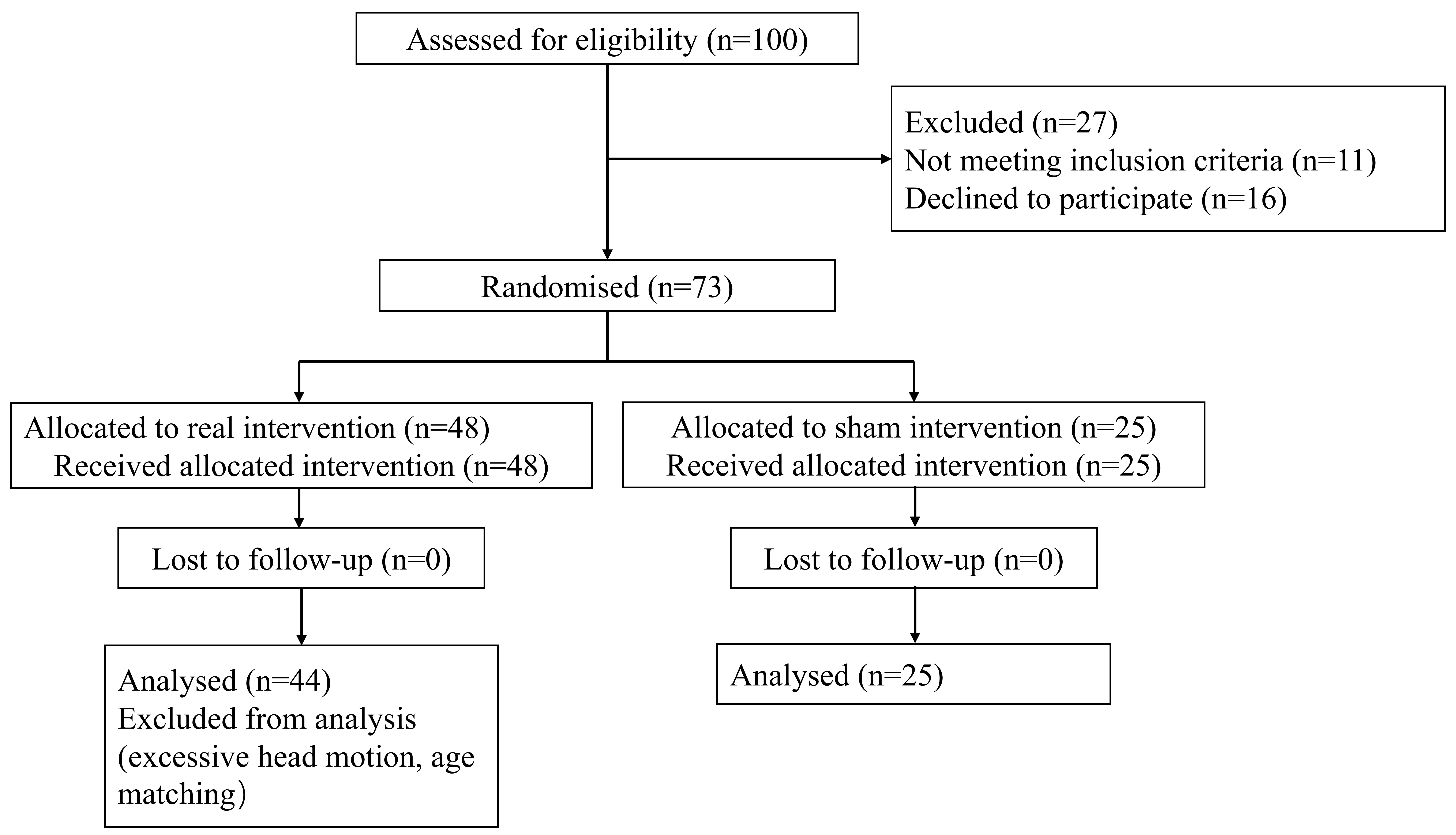


**Figure S1. The flowchart of participant enrollment, randomization, and analysis.**


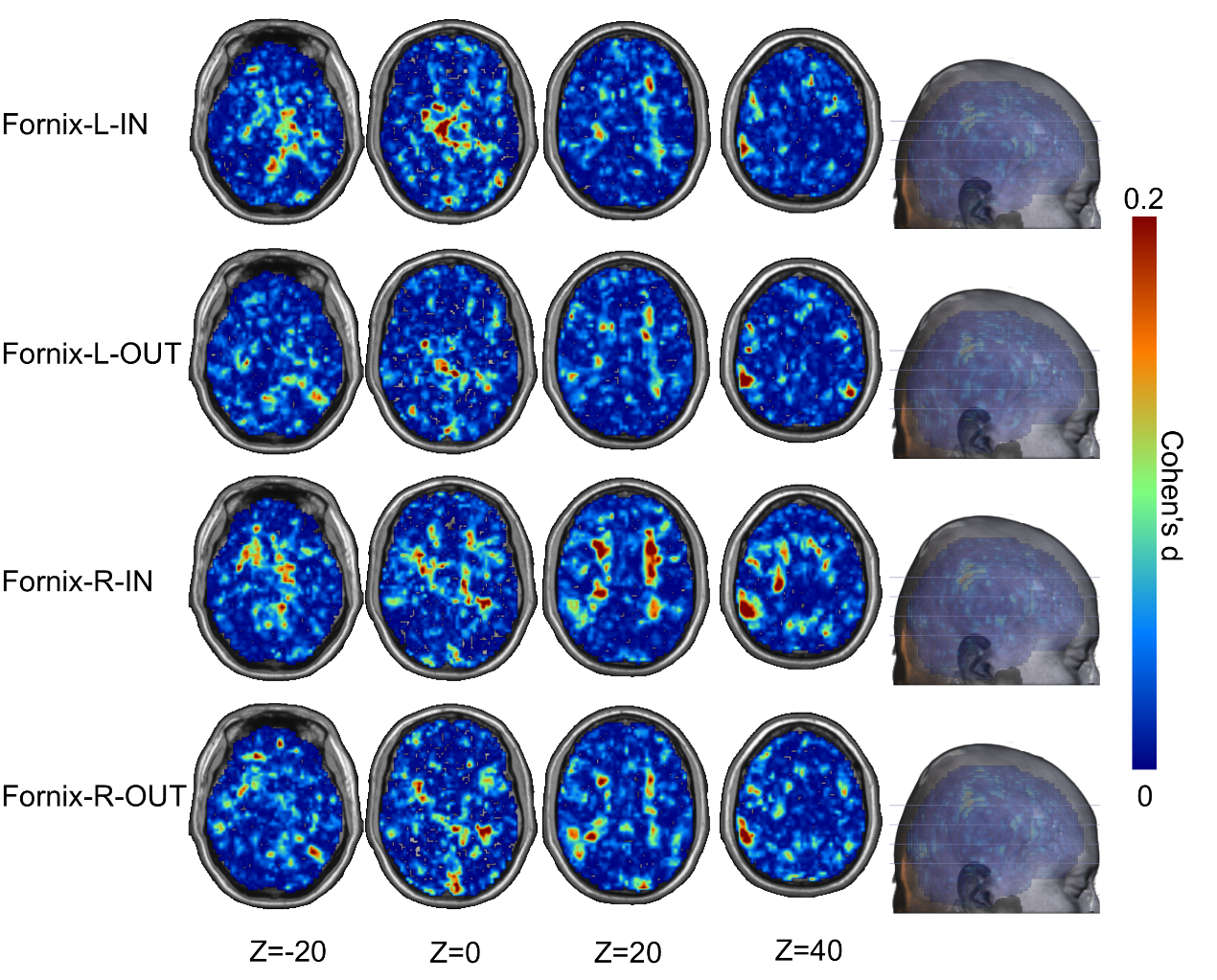


**Figure S2. Paired t-test Cohen’s d maps for pre- vs. post-treatment GCA maps in the fornix (left-in, left-out, right-in, right-out).**


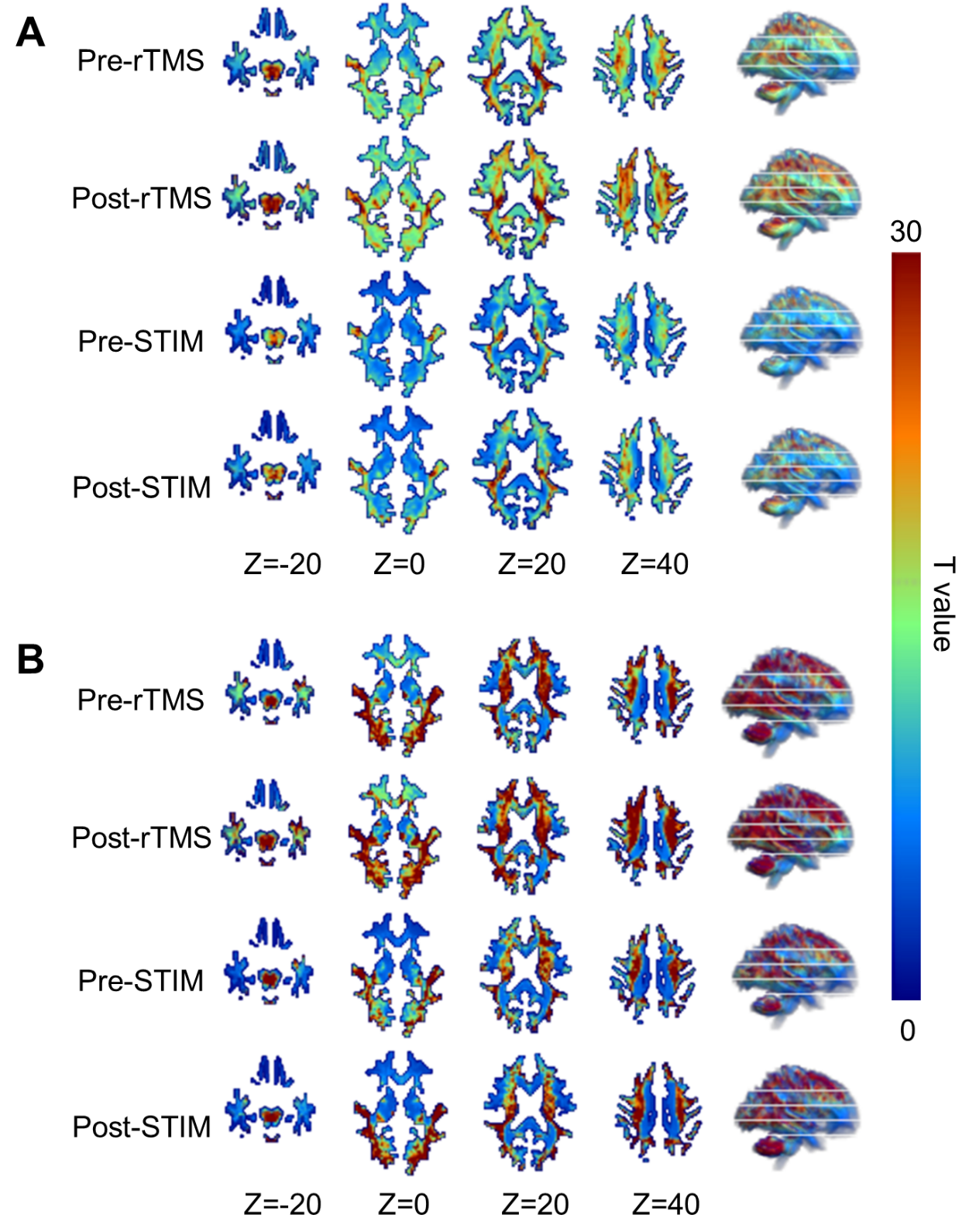


**Figure S3. One-sample t maps of DTI metrics.** (A) One-sample t maps of FA values within the four groups. (B) One-sample t maps of MD values within the four groups. STIM = stimulation.
